# Supplementary material for: Turbulent superstructures in Rayleigh-Bénard convection
Source: Nat Commun. 2018 May 29;9:2118. doi: 10.1038/s41467-018-04478-0 (PMC5974373; doi:10.1038/s41467-018-04478-0)
Supplement: Supplementary file 1 — Supplementary Information [file 41467_2018_4478_MOESM1_ESM.pdf]

Supplementary Information for

Turbulent superstructures in Rayleigh-Bénard convection

Ambrish Pandey[1], Janet D. Scheel[2] and Jörg Schumacher[1,+]

[1] Institut für Thermo- und Fluiddynamik, Technische Universität Ilmenau, Postfach 100565, D-98684 Ilmenau, Germany

[2] Department of Physics, Occidental College, 1600 Campus Road, M21, Los Angeles, California 90041, USA

[+] Corresponding author: joerg.schumacher@tu-ilmenau.de

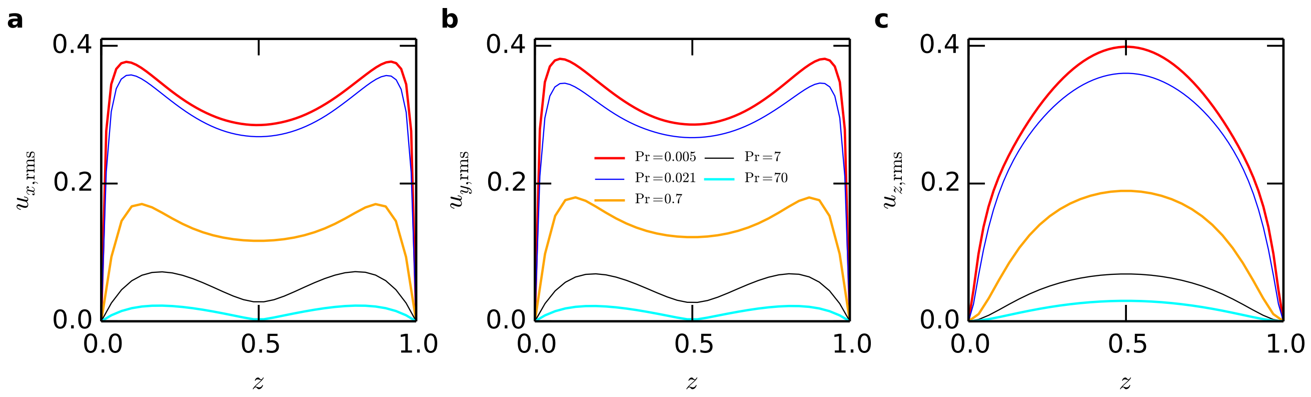

Supplementary Figure 1: Vertical profiles of the root mean square velocity fluctuations. The data are obtained by a combined temporal and plane average over the whole cross section area. (a) $u_{x,\mathrm{rms}}(z)$, (b) $u_{y,\mathrm{rms}}(z)$, and (c) $u_{z,\mathrm{rms}}(z)$. The corresponding Prandtl numbers are indicated in the figure. All data are collected at $\mathrm{Ra} = 10^5$.

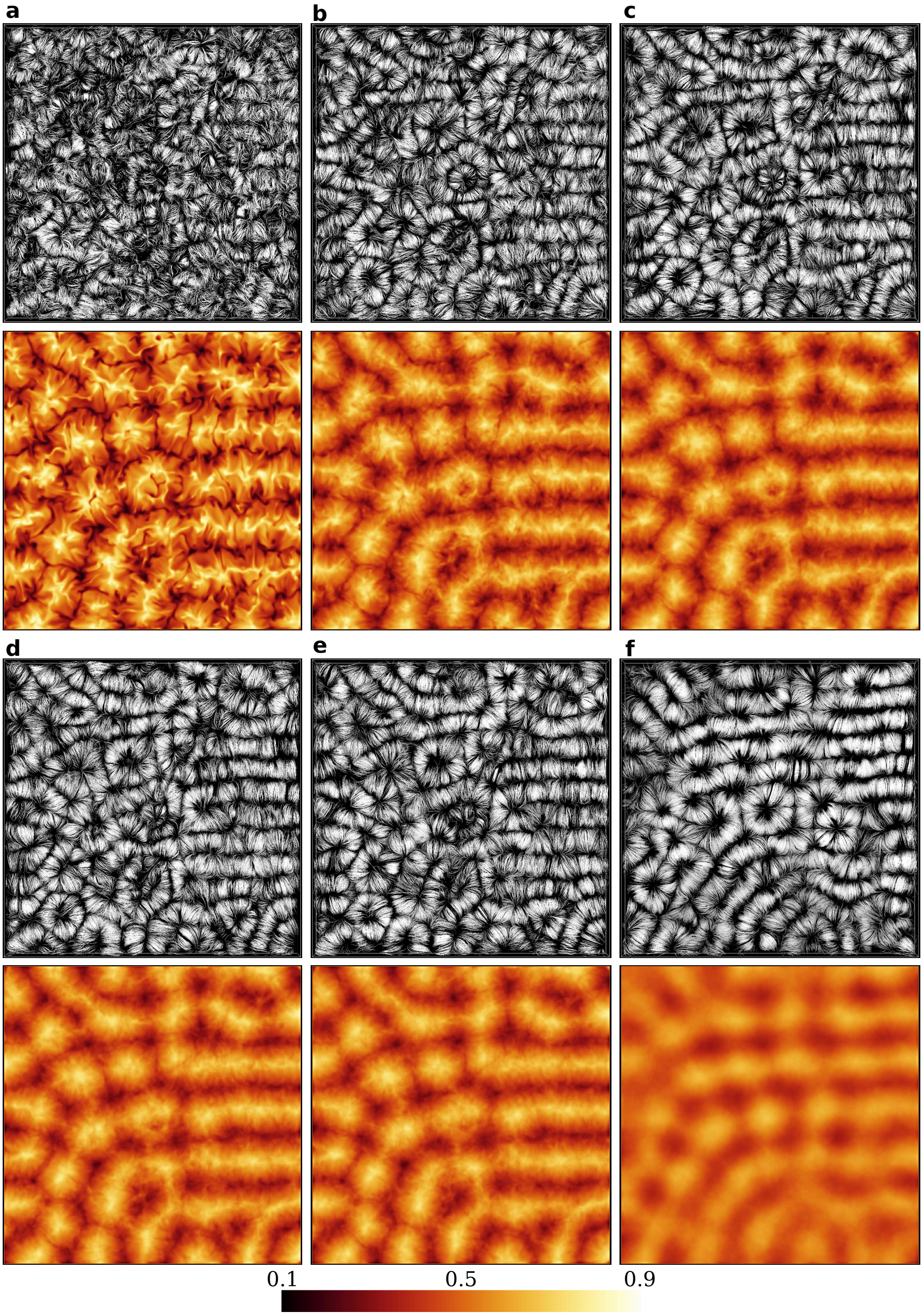

Supplementary Figure 2: Successively longer time averaging of simulation data. Results are shown for the simulation run at $\mathrm{Ra}=10^5$ and Pr=0.7. Velocity field (top) and temperature in the midplane (bottom) are displayed. (a) Instantaneous snapshot. (b–f) Time averaged fields for a successively longer interval length of $\tau/3$, $2\tau/3$, τ, $4\tau/3$, and 19.4τ. It can be seen that the patterns remain relatively independent of the interval length for panels (d) and (e) which corresponds to averages over 57 and 76 T_f, respectively. Panel (f) displays the time average over the full time integration.

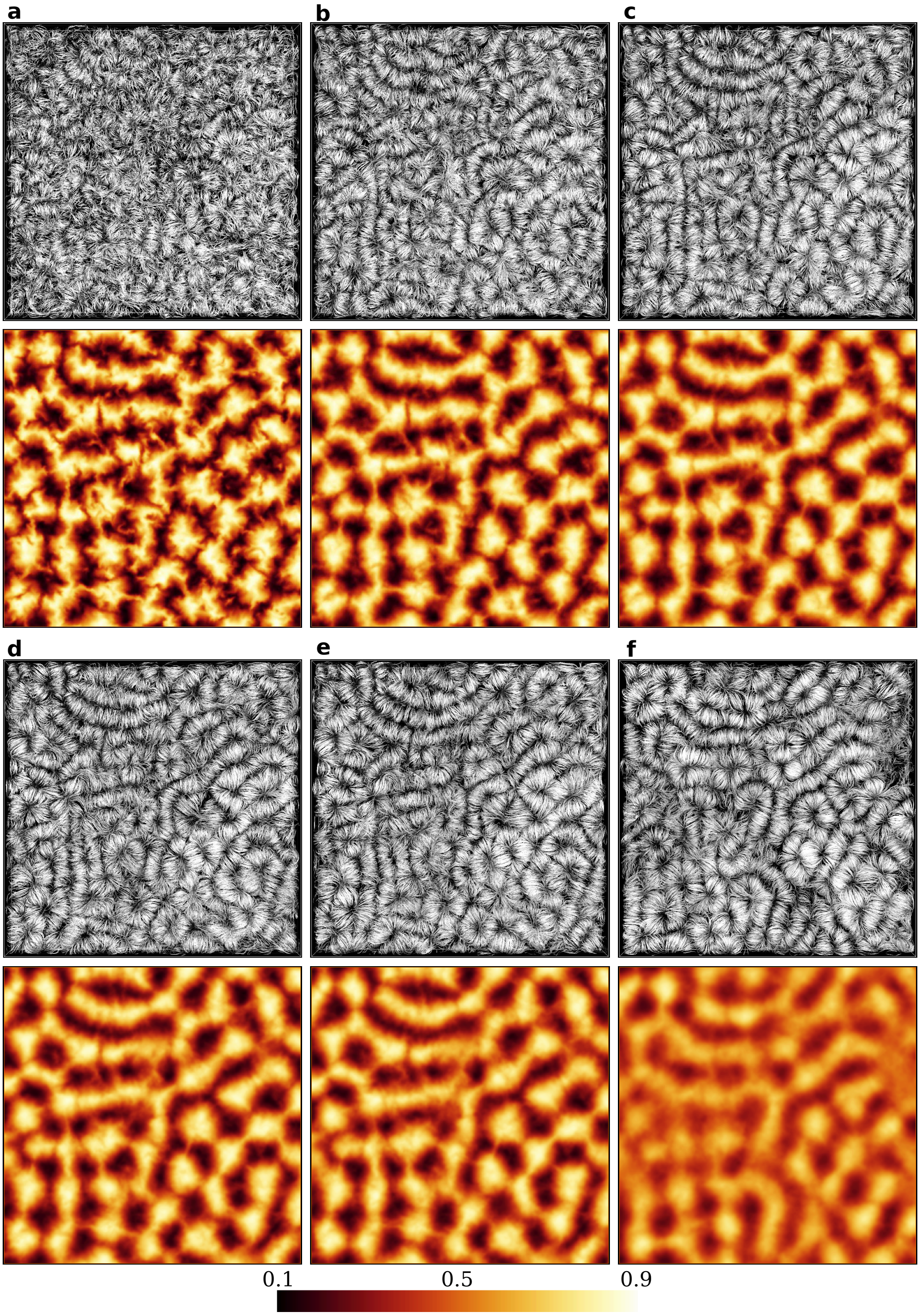

Supplementary Figure 3: Successively longer time averaging of simulation data. Results are shown for the simulation run at $\mathrm{Ra} = 10^5$ and $\mathrm{Pr} = 0.021$. Velocity field (top) and temperature in the midplane (bottom) are displayed. (a) Instantaneous snapshot. (b–f) Time averaged fields for a successively longer interval length of $\tau/3$, $2\tau/3$, τ, $4\tau/3$, and 5.5τ. It can be seen that the patterns remain relatively independent of the interval length for panels (d) and (e) which corresponds to averages over 27 and 36 T_f, respectively. Panel (f) displays again the time average over the full time integration.

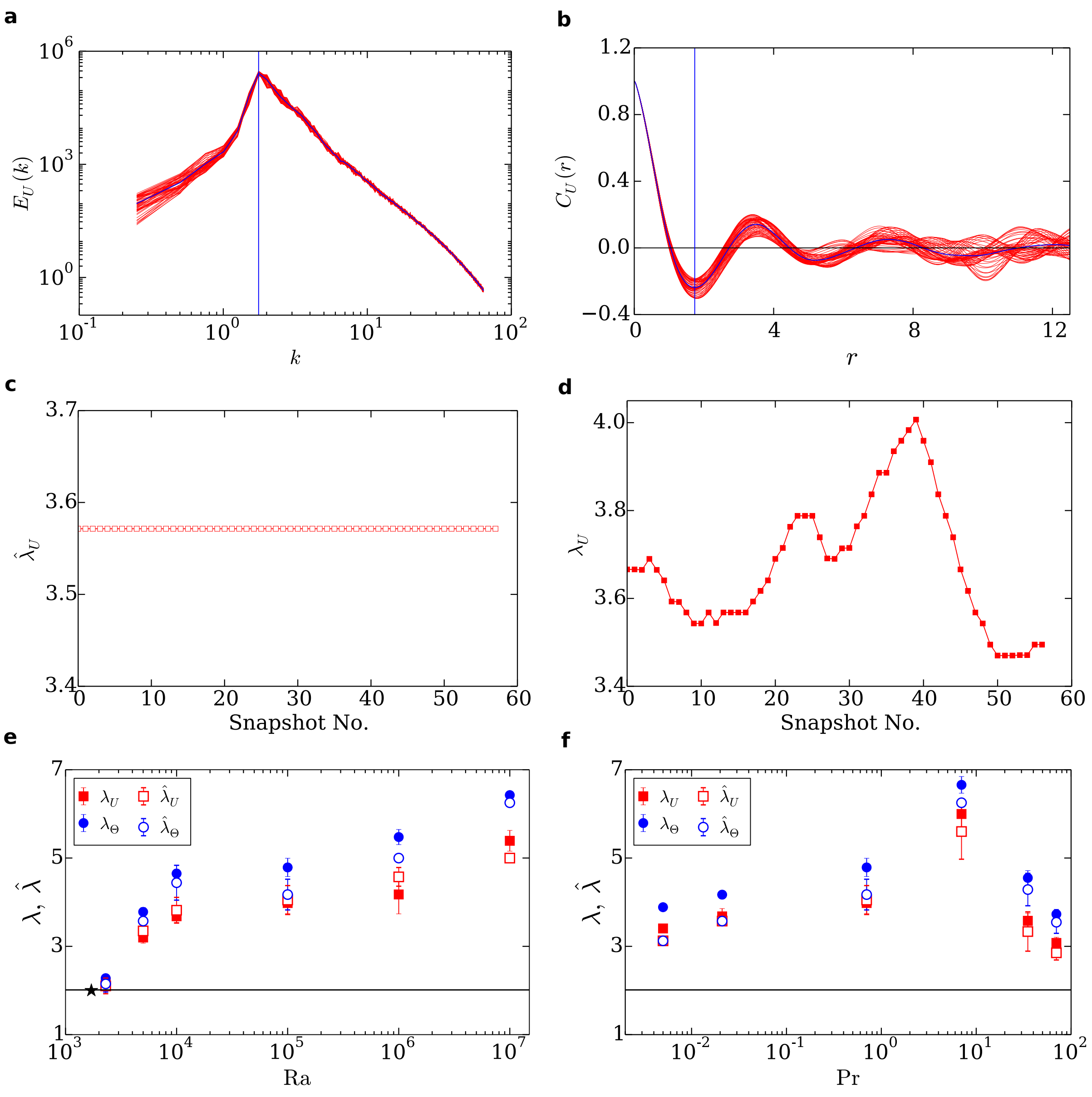

Supplementary Figure 4: Complementary scale analysis in physical space. Data are now analysed by means of correlation functions. Panels (a–d) show the data for one example run at Pr = 0.021 and Ra = 10^5. (a) Azimuthally averaged spectra of $\hat{U}(k_x, k_y; \tau, t_0)$ (red curves) and the mean over different t_0 of all these spectra (blue curve). The vertical solid line indicates the wavenumber that corresponds to the maximum of the averaged spectrum. (b) Spatial correlation functions taken for $U(x, y; \tau, t_0)$ in physical space along x-direction. Again, correlation functions at different t_0 (red curves) and the mean of all correlations (blue curve) are shown. The vertical solid line indicates the scale that corresponds to the minimum of the averaged correlation function. (c, d) Resulting characteristic scales of the superstructures, $\hat{\lambda}_U$ and λ_U versus snapshot number, i.e., as a function of t_0. (e, f) Summary of characteristic scales for U and Θ which are obtained in Fourier ($\hat{\lambda}$) and physical space (λ), respectively. The horizontal solid lines in panels (e) and (f) stand for the critical wavelength at the onset of convection. Star symbol in panel (e) stands for $\mathrm{Ra_c} = 1708$.

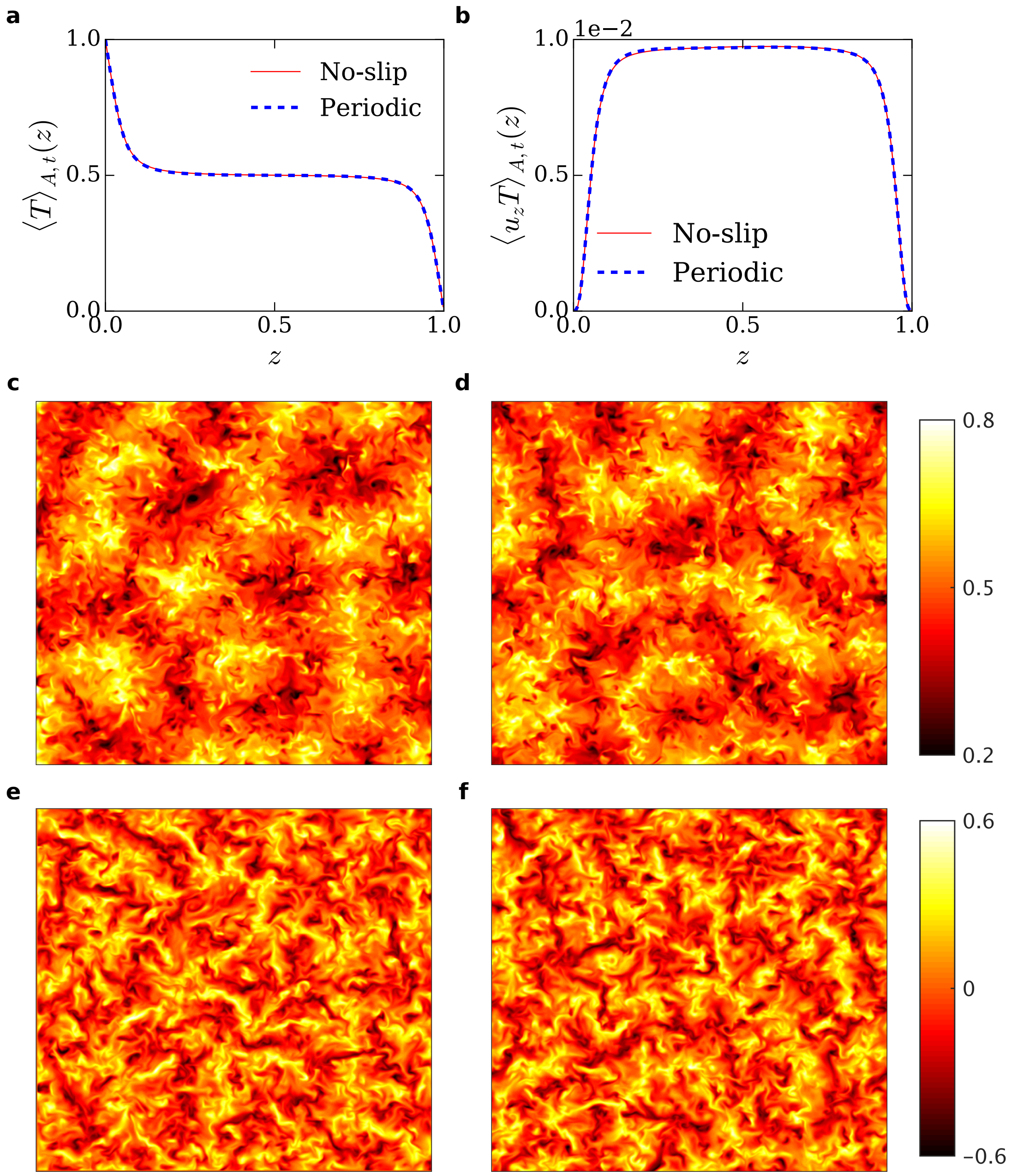

Supplementary Figure 5: Comparison between the cases with no-slip and periodic sidewalls. Results are for a cell with $\Gamma = 16$, $\mathrm{Ra} = 10^6$, and $\mathrm{Pr} = 0.7$. Vertical profiles of (a) horizontally- and time-averaged temperature and (b) convective heat flux. Contour plots of the instantaneous temperature field T in midplane for (c) closed box and (d) periodic box. Contour plots of the instantaneous vertical velocity component u_z in midplane for (e) closed box and (f) periodic box. Color bars to the right are taken for both panels in the middle and bottom rows.

Quantity	No-slip sidewalls	Periodic sidewalls
$u_{\rm rms}$	0.241 ± 0.001	0.245 ± 0.001
Re	288 ± 2	293 ± 2
Nu	8.1 ± 0.05	8.1 ± 0.04
$\hat{\lambda}_\Theta$	5.3 ± 0.0	5.3 ± 0.0
λ_Θ	5.4 ± 0.3	5.4 ± 0.4
$\hat{\lambda}_U$	3.4 ± 0.5	4.0 ± 0.0
λ_U	3.1 ± 0.6	3.2 ± 0.7

Supplementary Table 1: Comparison of a few important quantities for the cases with rigid no-slip and periodic sidewalls. The aspect ratio is $\Gamma = 16$, the Rayleigh number is $\mathrm{Ra} = 10^6$, and the Prandtl number is $\mathrm{Pr} = 0.7$.

Supplementary Note 1. Velocity fluctuations

Supplementary Figure 1 shows the profiles of the root mean square fluctuations of the three velocity components. They are given by

$$u_{x,\rm rms}(z) = \langle u_x^2(z) \rangle_{A,t}^{1/2} \,, \tag{1}$$

$$u_{y,\rm rms}(z) = \langle u_y^2(z) \rangle_{A,t}^{1/2} \,, \tag{2}$$

$$u_{z,\rm rms}(z) = \langle u_z^2(z) \rangle_{A,t}^{1/2} \,, \tag{3}$$

where $\langle \cdot \rangle_{A,t}$ denotes an average over horizontal $x - y$ planes at fixed z and time. It can be seen that the profiles for $u_{x,\rm rms}$ and $u_{y,\rm rms}$ agree well, as expected in a horizontally extended domain. The convection flow at $\mathrm{Ra} = 10^5$ is in a turbulent state for Prandtl numbers $\mathrm{Pr} \leq 0.7$. For $\mathrm{Pr} \geq 7$ the fields are in a chaotically and time-dependent state. The magnitude of all three profiles $u_{i,\rm rms}$ is significantly reduced for $\mathrm{Pr} = 70$ as seen in Supplementary Figure 1.

Supplementary Note 2. Superstructures as a function of the interval length of time average

As mentioned in the main text, the time averaging window should be long enough to remove small-scale turbulent fluctuations. The appropriate time scale is determined from the characteristic horizontal pattern scale and the amplitude of the turbulent velocity fluctuations (see Equation (7) of the main text). Both of these quantities are only determined after the simulation is finished. For each parameter set, we tested how the patterns are affected by a successively longer time average interval. Supplementary Figures 2 and 3 summarize these results for the runs at $\mathrm{Pr} = 0.7$ and 0.021, respectively. Panel (a) in both figures is an instantaneous snapshot, the averaging time interval is successively increased and centered around the original snapshot. Compared to panels (a) in both figures it can be seen that the patterns in panels (d) and (e) remain relatively robust. While the large-scale structures of the temperature are visible for shorter time intervals already, they become clearly observable for the velocity only after a certain length ($\sim \tau$) of the interval. For even longer time averages in turn, the temperature structures begin to become washed out while velocity patterns do not disappear, but change gradually.

Supplementary Note 3. Complementary pattern scale analysis in physical space.

Supplementary Figure 4 provides results that were obtained by a complementary analysis of the spatial correlation functions in physical space. Similar to the spectral analysis in the main text, we average the vertical velocity u_z and temperature deviation θ over a time interval τ and thus generate a sequence of fields $U(x, y; \tau, t_0)$ and $\Theta(x, y; \tau, t_0)$ at different times t_0 (see also Equations (4) and (5) of the main text). The correlation functions are given by (here along x direction)

$$C_\omega(r; \tau, t_0) = \frac{\langle \omega(x + r, y; \tau, t_0) \omega(x, y; \tau, t_0) \rangle_{x,y}}{\langle \omega^2 \rangle_{x,y}} \,, \tag{4}$$

with $\omega = \{U, \Theta\}$ taken at $z = 1/2$. Correlations along the y direction are defined similarly. A well-defined minimum of C_ω with strongest anti-correlation is found for all data sets at $r^*_{U,\Theta} = \lambda_{U,\Theta}/2$. Supplementary Figure 4(b) displays the obtained results for U. For completeness, we replot the corresponding spectra in Supplementary Figure 4(a) and indicate the maximum

$$k^*_U = \frac{2\pi}{\hat{\lambda}_U} \,. \tag{5}$$

The analysis for Θ proceeds in exactly the same way. The analysis along x and y directions is combined. Supplementary Figures 4(c,d) display the resulting characteristic scales obtained in Fourier space by means of the azimuthally averaged spectra and in physical space by means of correlations along x and y directions versus t_0 or starting snapshot number of time average window τ. The characteristic superstructure scales $\lambda_{U,\Theta}$ and $\hat{\lambda}_{U,\Theta}$ are found by averaging over all snapshots. The results are compared in Supplementary Figures 4(e, f). It is seen that the results for the characteristic scales which we obtained in two ways agree very well.

Supplementary Note 4. Comparison of periodic and rigid sidewalls

For large aspect ratios as discussed here, it can be expected that the effects of the sidewall boundary conditions become subdominant. In the following, we provide a comparison of two cases for an aspect ratio of $\Gamma = 16$ at a Rayleigh number $\mathrm{Ra} = 10^6$ and a Prandtl number $\mathrm{Pr} = 0.7$. We note that this aspect ratio is smaller than the one which is used throughout the work. It can thus be expected that differences, if existing, would be more obvious here. In Supplementary Figure 5, we display the mean vertical profiles of the temperature T and the convective heat flux $u_z T$ for both cases and do not detect any significant differences. Also the visual inspection of the instantaneous contour plots in the midplane does not reveal any clear differences in scales and amplitudes. Supplementary Table 1 lists further details of the comparison. It is shown that the root mean square velocity in the full volume, u_{rms}, the Reynolds number, Re, and the Nusselt number, Nu, agree. We also compared the characteristic scales of the superstructure patterns. The characteristic scales of the temperature field agree for both sets of boundary conditions when determined in physical and Fourier spaces. A slight difference is observable for the vertical velocity field component. While the physical space analysis reveals similar characteristic scales for both boundary conditions, the Fourier space analysis shows a difference. The smaller $\hat{\lambda}_U$ can be traced back to a variation of the maximum wavenumbers of the spectra in the no-slip case which is not observed for the periodic case where statistical homogeneity is present in x- and y-directions. The maximum wavenumbers taken for individual snapshots display partly larger values than for the periodic case. This results in a slightly smaller wavelength for the time average.
